# Hospital and Institutional News

**Published:** 1920-11-20

**Authors:** 


### HOSPITAL AND INSTITUTIONAL NEWS.

### THE CENOTAPH.

A second anniversary of the Great Armistice has passed, and with it some of the most impressive ceremonies it has been our privilege to witness. But even more lasting in the memories of those here in London than the homecoming of the honoured Unknown and the unveiling of the beautifully simple Cenotaph will remain the sight of the long, patient, unceasing processions of people reverently and spontaneously visiting the spot during the succeeding days. The memorial itself, banked high with platforms of flowers, receiving every minute some fresh increment, and surrounded by an ever changing but silent throng drawn from all parts of the country, has provided a spectacle which had to be seen to be understood. And there is to no one any possible doubt that, whatever its true artistic merits, in the Cenotaph and its ceremonies recently associated with it, the public have been able to recognise both a focus and a form of expression for some of their sincerest thoughts and the most sacred of their memories. The whole circumstance has brought to the surface a multitude of feelings, which by their real and obvious solemnity have more than disproved the accusation that the United Britain of the war period is lost and forgotten in the restless social upheavals which loomed so darkly but a few weeks ago. As our senior daily contemporary remarked, no nation, no metropolis, can be shown to possess a common consciousness, unless at some dedicated moment its spiritual unity takes visible form. Now again we see that underlying all the recently expressed discontent there still exists that sense of common fellowship which did so much to bring us victory in the cruellest of all wars. May we hope that this wonderful demonstration of the nation's true character and the innermost feelings of her people will have brought the light of understanding to not a few of our reckless domestic malcontents, who have so recently threatened the fruits of this very heroism which Britain holds so sacred.

### PROGRESS OF THE BILL.

The Ministry of Health (Miscellaneous Provisions) Bill entered the Committee stage on Monday night and was accompanied by profound apologies from its author and the leader of the House as to its complex nature. Some simplification was made by the offer of the withdrawal of thirteen clauses, ranging in character from testing clinical thermometers to purchase and appropriation of land. So far, however, the form of Clause 11, relating to municipal and voluntary hospitals, is not altered beyond the amendments during the second reading. The financial aspect of the measure secured most attention, and the Government promised some protection of the interests of ratepayers by the setting up of a Select Committee to consider in what way Parliament could effectually exercise some control on expenditure by the rates in relation to all Bills introduced into the House of Commons.

### MEDICAL RESEARCH.

On Armistice Day the formal presentation was made of the Royal Society of Medicine's Gold Medal to its first recipient, Sir Almroth Wright, and on this occasion he delivered also a particularly interesting address on medical research. Several sections of this speech are well worthy of detailed consideration, and next week we hope to publish them. For the moment, however, we must simply call attention to the text of his arguments. In the first place it was claimed that every hospital must have its own fully-equipped laboratory, otherwise treatment in our hospitals cannot efficiently progress. All research must be done in connection with hospitals and by a specially trained staff. Research services must be organised. Not only medical, but laboratory training, as such, is essential, and the speaker strongly advocated some form of staff college where this training can be obtained. The man taking up medical research as a career is at present in a far from assured position concerning his remote or immediate future, and the solution of this problem is one of the urgent questions of scientific medicine in this country.

### THE TASK OF TROPICAL MEDICINE.

At the close of last week the Duke of York formally opened the new premises of the London School of Tropical Medicine and the Hospital for Tropical Diseases at Endsleigh Gardens, Euston Road. The tributes which the royal visitor paid to the past work of this Institute were indeed well deserved, and, in view of the outstanding importance which this branch of medicine must hold in the future economy of the world, it is to be hoped that the Government will at all times spare no pains to see that a science, which is a truly Imperial consideration, shall receive adequate monetary support. We may be already proud of the system of tropical hygiene which the Briton has carried into so many lands, but as time goes on the civilised world becomes more and more dependent upon the products of these tropical regions, and it is the first duty of the nation, Government and people to see, during the discussion of so many home health questions, that tropical medicine does not suffer relative neglect.

### SHARP CRITICISMS OF THE BRADFORD MUNICIPAL HOSPITAL SCHEME.

As usual big municipal proposals for hospitals have not matured, in this case, at Bradford, without unsuspected complications. The local branch of the British Medical Association lately waited on Dr. Addison to protest that the Bradford Health Committee had not consulted the local profession in its plans, and that the only medical member upon the Health Committee was not chosen to accompany the various deputations which have waited on the Ministry of Health. Simultaneously the Committee of the Bradford Royal Eye and Ear Hospital has passed a resolution "viewing with alarm the proposal of the Bradford Health Committee to establish a department for the treatment of aural and ophthalmia cases at the public's expense, there being adequate accommodation and staff at this hospital to meet all requirements." It is also urged that it was misleading for the Health Committee to declare that a municipal hospital with 1,100 beds would be established; that the hospital, in fact, will not be a general hospital, because all the beds, except 100, are already ear-marked for other cases. Dr. Basil Hall, the Bradford surgeon, declares that the municipal hospital will continue to do the old Poor-Law work under the guise of á general infirmary. Mr. Hall argues, therefore, that the city will benefit very little by this ambitious-seeming scheme. At all events, high expectations have been encouraged. It is for the Chairman of the Bradford Health Committee to explain what really is in hand.

### THE VALUE OF HOSPITAL SITES.

Mr. Reginald M. Phillips, who is described as a young financier and estate agent, has views on the hospital situation. Amalgamation, he says, is the only way out; the cost of organisation to-day entails such heavy burdens that hospitals must find a way to economise. Banks amalgamate for reasons of economy. Many of the hospitals occupy sites in some of the most important positions in London, which are of much greater value for housing commerce than for hospital purposes. They should be re-established on sites of a more economical kind, and surplus capital would put them in funds for years to come. Look at St. George's Hospital. Look at possible sites in outlying districts. We do not say that Mr. Phillips is wholly right or wholly wrong. Certainly the idea is not bad for the estate-agent business, which does not interest us; but is it good for the hospitals, which does? The cost of rebuilding, for one thing, is deterrent; another consideration is that what from the point of view of prominence in geographical position is good for an hotel or a theatre is equally good for a hospital. One can hardly blame St. George's Hospital for sticking to a site which has brought it in the past hundreds of thousands in legacies and donations, and will bring thousands more which would not accrue to an institution stowed away in a back-water.

### NEW RADIUM HOSPITAL.

The Manchester Royal Infirmary has received a splendid gift from Sir Edward and Lady Holt, in the shape of the building lately used by the Nelson House Nursing Home, which is quite close to the infirmary, and which has been fully equipped by the donors as a hospital for the treatment of patients by radium. This will also relieve the Manchester Radium Institute, which has for some time been severely handicapped by its lack of accommodation and facilities to deal with the growing number of cancer and other malignant and skin diseases. There are thirty beds at Nelson House, and when it is opened it will be the first hospital in the United Kingdom exclusively for radio treatment. Although it will still be possible to treat patients at the other hospitals when the new institution is in full working order, the economy of a single centre, from the point of view of time, labour, and expense, will be great. The new institution will by arrangement work also as an auxiliary to several other hospitals in Manchester and outlying districts.

### THE FOOD PERIL.

Recently the Marylebone Borough Council decided to make representations to the Ministry of Health suggesting the desirability of steps being taken to require the licensing or registration by the local authority of premises where food is prepared or cooked, having regard to the present difficulties experienced in maintaining effective supervision of such premises. Dr. Charles Porter, the medical officer of health, has now called attention to the fact that the Wallasey Corporation, in view of similar difficulties, obtained power under a local Act, passed this year, to forbid owners or occupiers of premises used for the preparation or manufacture of potted or preserved meat, fish, or other food intended for the purposes of sale, using such premises until they had been registered, and when necessary to refuse registration. It is, therefore, now proposed to ask the Health Ministry to obtain for all local authorities powers similar to those provided in the Wallasey Corporation Act. Certainly, if supervision of food premises is needed in Wallasey, it is needed everywhere else.

### A CHOCOLATE PERIL.

Evidently there is a risk to health in eating chocolates, which are not always wholesome. The Hammersmith Borough Council reports that in consequence of complaints the medical officer of health seized 60 lb. of chocolates from a certain shop in the district. The chocolates were subsequently condemned as unfit for human consumption, being infested with the larvæ of a certain species of moth, known in connection with the manufacture of chocolates and biscuits, but which are said not to be dangerous to health. In some instances the seized chocolates were infected with mould. The Council has warned the manufacturer and retailer of the chocolates that if a similar case is discovered, proceedings will be taken against them for exposing for sale food which is unsound, unwholesome, or unfit for human consumption.

### A "LIFEBOAT" AMBULANCE.

The account of a lifeboat having been used as an ambulance for conveyance by water is probably unique. It appears that a young man in the Scilly Isles was suffering from an acute disease necessitating removal to Penzance for immediate operation. On account of the rough weather, however, it was impossible for the local steamer to make the crossing; the lifeboat therefore was requisitioned, and the patient safely transferred to the mainland.

### NEW INTERNATIONAL JOURNAL.

The title of this newly-born publication is *The International Journal of Psycho-Analysis*, edited by Dr. Ernest Jones, who explains that its purpose is to cater for English readers of this subject as is done by various journals in America. We should like to send copies to Mr. Hilaire Belloc and his fellow-romantic Mr. G. K. Chesterton. Freud himself leads off with a simple, short, illuminative article on the great obstacle to understanding psycho-analysis—namely, the recognition of unconscious mental processes. Then follows a not very convincing pathography of Henry the Eighth (it would not be with this that Mr. Belloc would quarrel, however). Dr. Douglas Bryan begins a series of elementary didactic articles on psycho-analysis; and an English chemist has a bibliography of recent literature. Reports of meetings and a review of the subject close the number. We notice the name of an eminent English psychologist, Dr. W. H. R. Rivers, among the associate members. The publishers are the International Psycho-analytical Press, London, Vienna, and New York; and the subscription is 30s. yearly, or 18s. for members of the International Psycho-analytical Association. All English students of the subject who are unable to read German will welcome this new journal, which is of interest not only to medical men, but also to intellectual workers of various kinds.

### A HOSPITAL ADMINISTRATOR AT OXFORD.

We are glad to hear that success has attended the efforts to increase weekly contributions from the city and rural districts of Oxford for the Radcliffe Infirmary. Committees have been formed in thirty-four villages; thirty-one firms have started weekly contributions in Oxford, and already £770 has been received. The Sportsmen's Fund is also growing. Along with these beneficent financial changes, the administration has been concentrated in fewer hands. The committee system has been found cumbersome, and an administrator has been appointed, with a small committee, consisting of the Treasurer, the Rev. G. B. Cronshaw, the Vice-Chairman, and four members elected by the committee of management. The latter will serve for three years, after which they will not be eligible for re-election until a year has intervened. The Committee will meet weekly for routine business and to supervise the administrator's work. Besides the monthly meetings, it will have power to act on the reports from sub-committees and from the secretary of the medical staff. The new Committee's powers are limited to the spending of £50 on new work. It will present a written report to the monthly meetings of the board.

### STREET ACCIDENTS AND POOR-LAW INFIRMARIES.

Efforts have constantly been made to induce the authorities, more especially the police, to recognise the necessity for taking people to the nearest infirmary instead of removing them long distances to a hospital when they meet with a street accident. In one case an old lady was knocked down within three minutes' walk of the Lambeth Infirmary, where she was attended in the street by the infirmary medical staff. She was, however, removed to St. Thomas's Hospital by the police, who informed Dr. Baly, the medical superintendent, that their instructions were to take patients to the hospital. The strange part was that after the patient had been treated at the hospital she was sent back to the infirmary. Another effort is to be made to induce the responsible authorities to recognise that it is essential in a patient's interests that they should be admitted to a Poor-law infirmary close to where they meet with an accident rather than take them long distances, only probably to have to come back to almost the same place where the accident occurred.

### THREE THOUSAND MORE BEDS AT MANCHESTER.

The Manchester Board of Guardians is considering whether it can transform the Withington institution into an infirmary for all classes. The classification committee has been going into the question, and it is thought that if the necessary alterations were made, the institution could accommodate 3,000 patients. In view of the heavy waiting-lists at the voluntary hospitals of Manchester, this additional number of beds would be a great boon. Dr. Addison is already passively supporting those guardians who are already admitting paying patients; and the number of vacant beds in Manchester's Poor-law institutions has been put at 1,000, which, even if exaggerated, is an indication of the resources available.

### COVENTRY WORKERS EXCEL THEMSELVES.

It is announced that the special appeal to the workers of Coventry on behalf of the hospitals earlier in the year has resulted in a collection of £15,000. The contributions have been increased in some cases to 4d. a week, and in other cases to 1d. in the £ earned in wages. In no case, we believe, is the contribution now less than 2d. a week. The appeal for a special levy of 1s. for adults and 6d. for children, for one week, was also successful, and a sum of £1,250 was thus subscribed. The whole of the shop assistants are expected to become subscribers; and a number of small firms have also begun collections. The net result is best seen by the fact that last year's total was £8,350, a sum which has been nearly doubled. It is also noteworthy that the recent depression in trade adversely affected the total, so that we may hope in this case that what began as a special effort may be maintained, perhaps even surpassed, in future years. The Coventry Saturday Committee deserves the warmest thanks for its splendid work.

### NEW DIETARY FOR POOR-LAW INSTITUTIONS.

The new dietary Order issued by the Ministry of Health will be heartily welcomed by all progressive boards of guardians. It had long been felt that grave injustice was done to the inmates of the institutions by the guardians being forced to adhere strictly to a confined dietary table that allowed them no latitude. Under the new Order they will be able to vary the meals a great deal more, and, what is more important, be able to give a supplementary meal between the abnormal long wait from tea at five o'clock at night to breakfast at half-past seven next morning. This innovation is connected with the action of the Southwark Board of Guardians. During the war they decided to give their inmates each night a portion of boiled rice to supplement the reduced rations of the day. The war over, they applied to the Ministry of Health to continue the practice, but it was only after a vigorous fight that they obtained the required consent to give the additional rice, which has since been varied by other milk puddings. By the new Order they will be able to give the inmates a more substantial meal, and no time will be lost in taking advantage of the privilege.

### LADS' SANATORIUM TO BE OPENED AT HALE.

A want that has long been felt in combating the white scourge has been a sanatorium for lads from fourteen to eighteen years of age, the unsuitability of their having to enter a men's institution on reaching fourteen having been recognised. This want is now in progress of being provided for through the agency of the Church Army, who have already sanatoria for children, and who have been asked by the Ministry of Health to take up this department. A splendid establishment, approved by the Ministry of Health, at Hale, Hants, has been generously given by the Bishop of Winchester to the Church Army, and necessary alterations and equipment are in course of progress; but expenses are very heavy. Miss Walker, Hon. Secretary Medical Mission, Church Army, Bryanston Street, W. 1, would most gratefully acknowledge any gifts in money or in kind to help in the struggle against this disease.

### A NEW BRISTOL MATERNITY HOSPITAL.

A Maternity Hospital committee in Bristol has acquired 7 Brunswick Square, which was previously in use for institutional purposes, and it is hoped thus to relieve the need of maternity cases in the city, too many of which have to be nursed inadequately at home, even if the mothers are lucky enough to find house-room at all. In Brunswick Square twenty-two beds are available at an inclusive fee of thirty shillings a week. This, we understand, includes medical treatment and nursing. The honorary secretary is Mrs. W. C. H. Cross, and the new institution is supplementary to the scheme for maternity cases at the Royal Infirmary, which could not suffice to meet the need. The Mother School at Bedminster, which, so to say, is the stem from which the Brunswick institution has sprung, is already overstocked with patients, and has to refuse many applicants. It is hoped that the movement will be further extended, for the need is still great.

### INSTITUTION ISOLATED AT EDINBURGH.

The isolation of an entire institution has been successfully arranged at Edinburgh, where an outbreak of smallpox occurred in the middle of October among the inmates of Craiglockhart Poorhouse. All the patients had been infected from some common source; five were displaying symptoms, and these were detected in different parts of the institution. Dr. Williamson, therefore, promptly isolated the whole place, closed it to all newcomers, and confined the inmates within the precincts. This isolation, of course, included the officers. The institution was disinfected throughout, and 900 officers and inmates were vaccinated. The period of quarantine lasted till the beginning of November, when Dr. Williamson was convinced that no fresh cases were likely to occur. Those who had been discharged a few days before the outbreak were traced to their homes and removed to the reception house of the city. They also were detained for a fortnight, and the patients suffering from the disease have since recovered. This quarantine of a big institution was a large order; but the results seem entirely to have justified Dr. Williamson's drastic measures.

### EAST HAM'S NEW SANATORIUM.

"The Harts" at Woodford, which was recently purchased by the East Ham District Council, has now been opened as their tuberculosis sanatorium. The premises, which are extensive and stand in some thirty acres of ground, and were secured for £10,000, have been thoroughly overhauled and adapted for their present use at a cost which it is estimated will probably total £20,000. At the present time the institution consists of a main building, in which on the ground floor are two large wards for advanced cases; the first floor contains a large women's ward, with a number of smaller ones for children and individual cases, the matron's, senior nurses' and nurses' sitting-rooms, and on the upper floor is the accommodation for the night nurses, probationers, and domestic staff. In addition to this main building a large shelter has been erected, which, together with a number of huts, forms a separate block, while complete laundry, sterilizing, and power equipment has been installed, and the large conservatory adapted for the patients' use. A good deal of opposition was originally made by local residents to the establishment of the sanatorium at Woodford, but as the result of an inquiry held by the Ministry of Health official sanction was given to the scheme.

### THIS WEEK'S DRUG MARKET.

The market is still waiting for a revival of demand, but there are at present no signs that it is imminent. Holders of stocks are more than ever disposed to realise their holdings, even if it means a considerable reduction in prices, but the difficulty is to find buyers even on these terms. Under the circumstances quotations are in very many cases merely nominal. Potassium bromide has again receded in price, and can now be bought at one-fifteenth the figure at which it was quoted at one time during the war. This, however, is an isolated case, for in the majority of instances the downward movement has been slow and gradual. Owing to the poorness of the Spanish crop of saffron prices are higher. There is a rather better demand for cod-liver oil, and prices are steadier. Menthol, Japanese peppermint oil, and camphor are very quiet, but without noteworthy change in price. Permanganate of potash is in plentiful supply, and the price tendency is downwards. Practically all the synthetic drugs are extremely dull, with an easier price tendency.

